# Isolated right coronary lesion and anterolateral papillary muscle rupture - case report and review of the literature

**DOI:** 10.1186/1749-8090-7-75

**Published:** 2012-08-16

**Authors:** Dime Stefanovski, Asnat Walfisch, Saško Kedev, Salis Tager

**Affiliations:** 1 Cardiac Surgery Department, ACIBADEM Sistina Clinical Center, Skupi 5a, Skopje, 1000, Macedonia; 2 Hillel-Yaffe Medical Center, Hadera, Israel; 3 University Clinic of Cardiology, Medical Faculty, Skopje, Macedonia

**Keywords:** Cardiac catheterization/intervention, Coronary artery bypass grafts, Coronary artery imaging, Mitral valve, Myocardial infarction

## Abstract

Ischemic rupture of the anterolateral papillary muscle is uncommon due to its dual blood supply. It usually follows an ischemic event involving branches of the left circumflex or left anterior descending arteries. We present a case of a patient admitted with an acute inferior wall myocardial infarction and an isolated distal right coronary artery occlusion. Acute mitral regurgitation with rupture of the anterolateral papillary muscle was diagnosed on the fifth post-infarction day. The patient underwent mitral valve replacement and coronary artery bypass grafting to the posterior descending artery. We conclude that anterolateral papillary muscle rupture may also result from an isolated right coronary lesion.

## Background

Myocardial ischemia and infarction may lead to different papillary muscle involvements including prolapse, elongation or rupture. Papillary muscle rupture is a devastating complication with extremely poor natural prognosis [1].

The posteromedial papillary muscle has, in most cases, a single blood supply from the posterior descending branch of a dominant right coronary artery (RCA). Rupture of the anterolateral muscle is less common, occurring in only 25% of papillary muscle rupture cases. The reason for this relative rarity is its dual blood supply: from the first obtuse marginal artery, originating from the left circumflex artery (LCx), and from the first diagonal branch, originating from the left anterior descending artery (LAD) [2-4]. Papillary muscle rupture is optimally diagnosed by transesophageal echocardiography with high sensitivity and specificity.

Patients with this condition usually undergo urgent surgical intervention with associated high mortality rates. Timing of such intervention is dictated by the patient’s hemodynamic status. Once a ruptured papillary muscle is diagnosed, mitral valve surgery is best performed as soon as possible. Hemodynamic instability may necessitate insertion of an intra-aortic balloon pump as a bridge to surgery [5].

We present a case of acute anterolateral papillary muscle rupture, 5 days post myocardial infarction, in the presence of an isolated distal RCA occlusion.

## Case presentation

A 63-year-old male patient presented in the state hospital emergency room with chest pain, dyspnea and general weakness lasting for several hours prior to his admission. Past medical history was remarkable for hypertension, dyslipidemia, and non-compliance. The patient was a non-smoker, with a negative family history for coronary artery disease.

Vital signs upon admission included an irregular heart rate of 40 bpm and a blood pressure of 95/70 mmHg. Heart and lungs examination was unremarkable. No murmur was appreciated and the lungs were clear.

**Figure 1:**
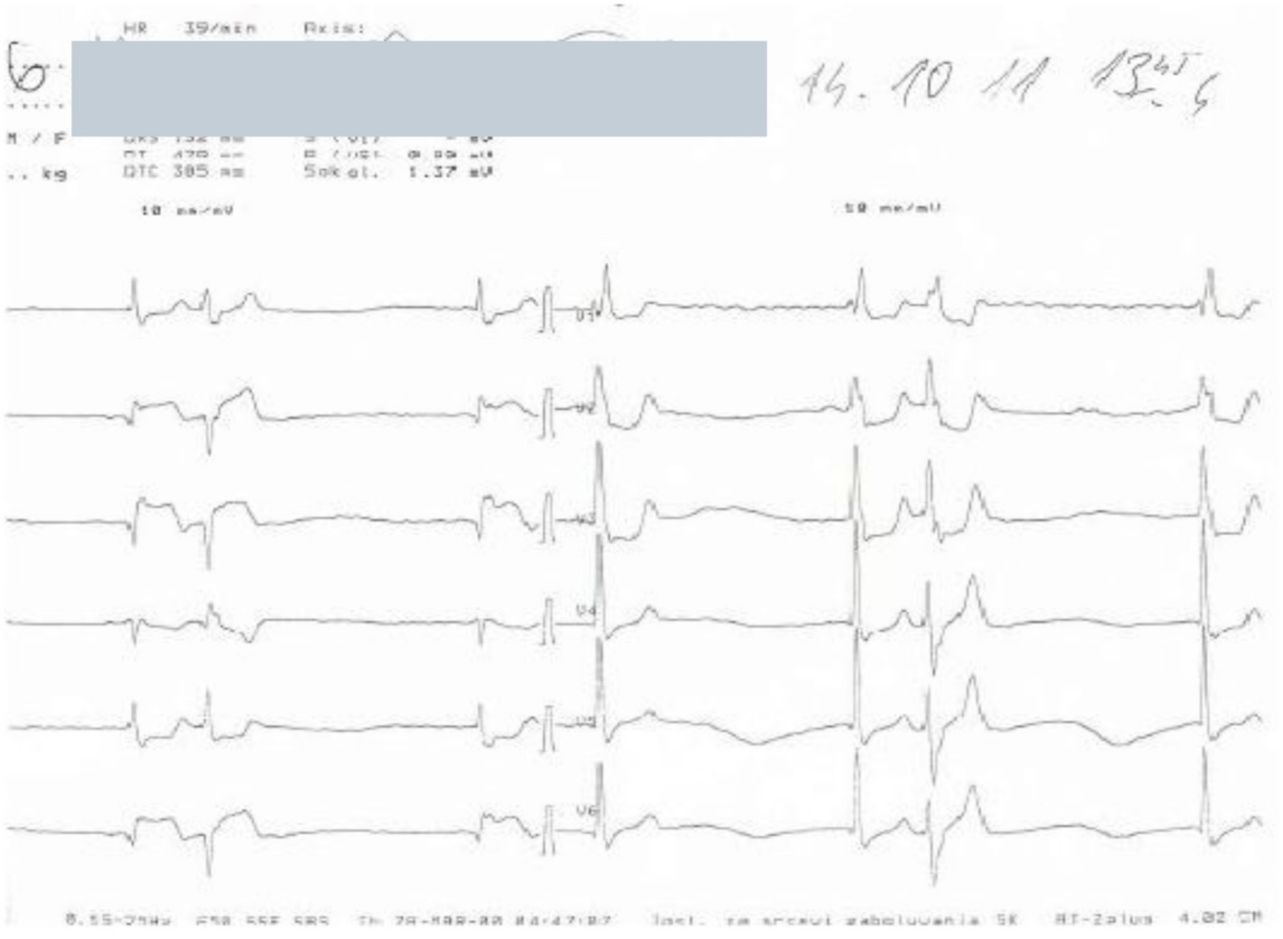
ECG upon admission.

ECG upon admission demonstrated Right Bundle Branch Block (RBBB) with atrial fibrillation, ST-segment elevation in leads TII, TIII, and AVF and ST depression in leads TI, AVL, V1-V4 (Figure 1).

Laboratory results revealed CK=4416 U/L and CK-MB=281 U/L.

**Figure 2:**
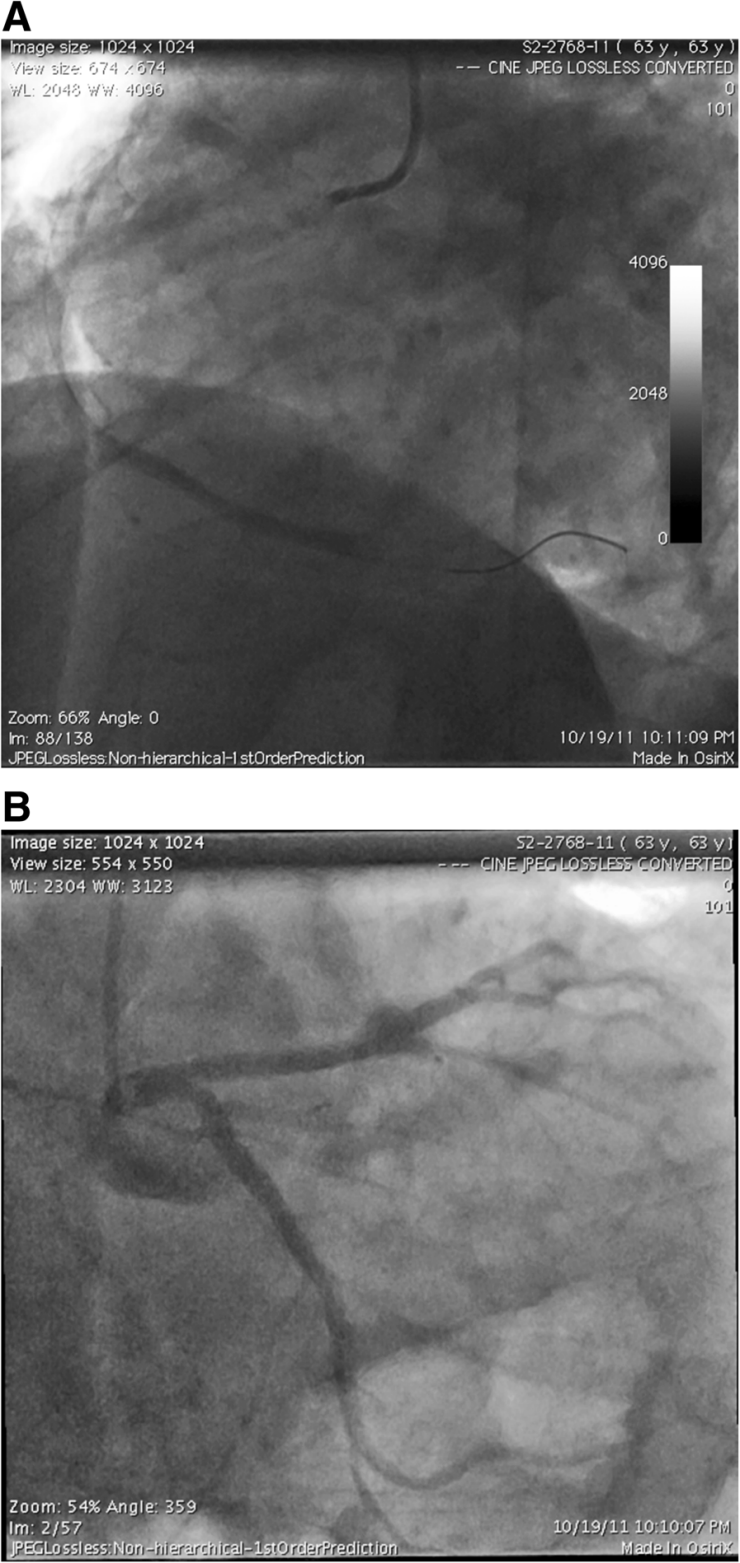
**Coronary angiogram of the: A. Right Coronary Artery showing complete occlusion of the distal RCA.B.** Left Coronary Artery showing normal anatomy.

**Figure 3:**
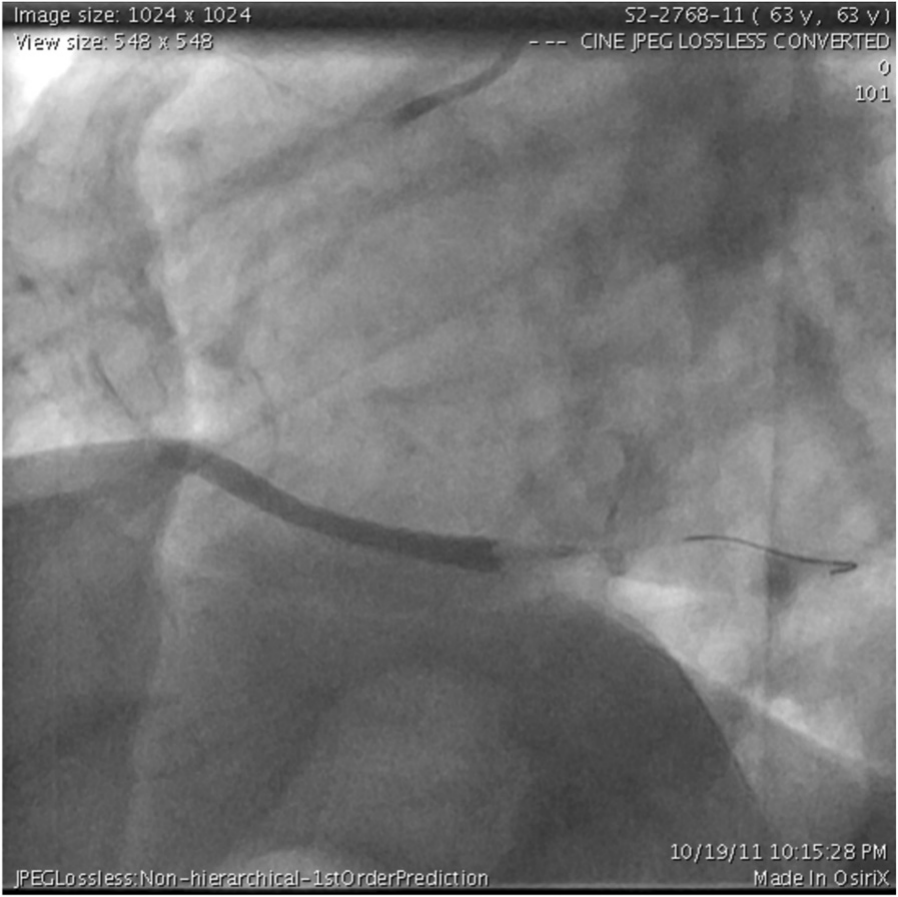
Right Coronary Artery showing complete occlusion of distal RCA following the failed balloon angioplasty attempt.

A coronary angiography demonstrated an isolated complete thrombotic distal RCA occlusion (Figure 2). The patient underwent an emergency percutaneous transluminal coronary angioplasty (PTCA) followed by a failed attempt of balloon angioplasty in the distal RCA (Figure 3).

On the fifth hospitalization day the patient’s condition suddenly worsened.

He become agitated and disoriented and showed signs and symptoms of heart failure. A transthoracic echocardiography revealed ejection fraction of 40% and a ruptured anterolateral papillary muscle with severe mitral regurgitation. Left atrial area was 24 cm^2^, the LV systolic volume was 92 ml and the LV diastolic volume 143 ml. The patient was immediately transferred to our institution and admitted to the cardiac surgery department for an emergency procedure. Upon admission, the patient was in cardiogenic shock, anuric, with a blood pressure of 70/50 mmHg and a heart rate of 200 bpm.

**Figure 4:**
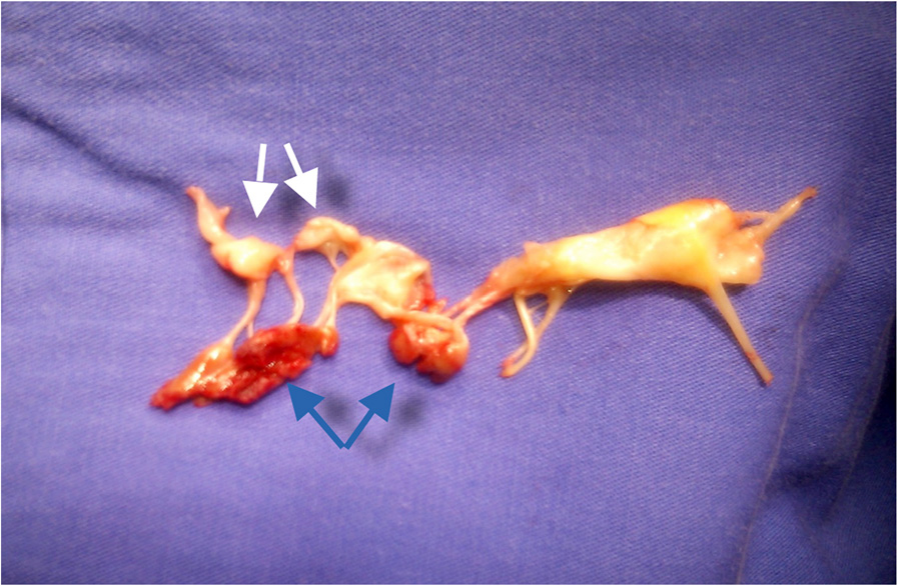
**The excised mitral valve showing complete rupture of the papillary muscle.** Blue arrows pointing to the ruptured papillary muscle head. White arrows pointing to anterior leaflet segments A1 &A2.

Cardiopulmonary resuscitation was commenced and an intra-aortic balloon pump inserted. Intraoperative findings included a complete rupture of the anterolateral papillary muscle head creating free mitral regurgitation (Figure 4). Leaflet segments A1 and A2 were completely flail. The mitral valve was replaced (Hancock 29 mm) and concomitant coronary artery bypass grafting to the PDA with a saphenous vein graft was preformed.

The postoperative course was remarkable for several hemodynamic instability episodes. On the sixth postoperative day, the patient developed bilateral pneumonia with a resistant strain of Staphylococcus Aureus. The patient’s status deteriorated, and he became gradually unresponsive to antibiotics and vasopressor therapy.

On the 9^th^ postoperative day the patient developed acute kidney failure (treated with continuous hemofiltration) and on the 12^th^ postoperative day, the patient died, from septic shock.

## Discussion

We present a case of acute rupture of the anterolateral papillary muscle, secondary to occlusion of the distal RCA. The ruptured papillary muscle resulted in severe free mitral regurgitation and cardiogenic shock. The ruptured papillary muscle appeared on the 5^th^ post-infarction day and was diagnosed with transthoracic echocardiography pre-operatively.

This case is significant due to the rarity in which isolated distal RCA occlusion results in ischemia and rupture of the anterolateral papillary muscle.

The clinical presentation of papillary muscle rupture depends on the extent of myocardial damage and severity of the mitral regurgitation (MR). It is usually clinically apparent around 5 to 6 days following the acute MI [6]. In our case the acute MR and corresponding cardiogenic shock appeared on the fifth post infarction day.

As mentioned above, the anterolateral papillary muscle is less often involved in a rupture compared with the posterior papillary muscle, because of its dual blood supply. However, our case provides clear proof of a distal RCA occlusion, with no involvement of the left coronary artery or its branches, leading to a rupture of the anterolateral papillary muscle.

To the best of our knowledge this is the first reported case of such clinical sequence. Previous reports have found that rupture of the anterolateral papillary muscle followed an obstruction in the LCx, LAD [6-8], or in one of the diagonal branches [4] but not in the RCA.

We believe that an overwhelming right predominance of the blood supply to the anterolateral papillary muscle led to this catastrophic outcome. Nevertheless, the angiogram of the left system shows a rather large circumflex artery, suggesting normal coronary artery anatomy. Thus, an alternative explanation is also possible: Since the patient entered the hospital in atrial fibrillation it is possible that the RCA occlusion was embolic. If so, one embolus could also have gone to a branch feeding the anterolateral papillary muscle from the left side.

The presence of a ruptured papillary muscle and severe MR ***prior*** to the presented ischemic event is highly unlikely due to the following: Nothing in the history of the patient was suggestive of a prior significant valvular disease and no heart murmur was appreciated upon admission to the hospital. Additionally, the echocardiographic appearance and measurements following the patient’s acute deterioration were suggestive of acute MR rather than a long standing MR.

In this case the extent of the infarction, the failed balloon angioplasty, the severity of the MR, and the later development of severe unresponsive sepsis, led to the patient’s death.

## Conclusion

Our case demonstrates that an isolated lesion of the distal RCA may result in rupture of the anterior papillary muscle. This clinical sequence may be explained by either an overwhelming right predominance of the blood supply to this papillary muscle or an embolic event.

## Consent

A written informed consent was obtained from the patient’s next of kin for publication of this case report and accompanying images. A copy of the written consent is available for review by the Editor-in-Chief of this journal.

## Abbreviations

RCA, Right coronary artery; LCx, Left circumflex artery; LAD, Left anterior descending artery; BPM, Beats per minute; ECG, Electrocardiogram; RBBB, Right bundle branch block; CK, Creatine kinase; CK-MB, Creatine kinase muscle –brain; PTCA, Percutaneous transluminal coronary angioplasty; PDA, Posterior descending artery; MR, Mitral regurgitation; MI, Myocardial infarction.

## Competing interests

The authors declare that they have no competing interests.

This case report was not funded. The authors of this paper had full control of the design of the study, methods used, outcome parameters and results, analysis of data and production of the written report.

## Authors’ contribution

DS has participated in the management of the patient and has written most of the manuscript. AW has conceived of the study and participated in drafting and finalizing the manuscript. SK has participated in the management of the patient, interpretation of the different test results, and drafting of the manuscript. ST has participated in conceiving of the study, patient management, coordination and review of the manuscript draft. All authors read and approved the final manuscript.

## Authors’ information

ST – Is the head of the Cardiac Surgery dept. at the ACIBADEM Sistina clinical center where the patient was operated on. He is also a professor at the Ss. Cyril and Methodius University Medical Faculty in Skopje, Macedonia.

SK – Is the head of the University Clinic of Cardiology in the State Clinical Center where the patient was catheterized and initially managed. He is also a professor at the Ss. Cyril and Methodius University Medical Faculty in Skopje, Macedonia.
